# Use of Electronic Auscultation in Full Personal Protective Equipment to Detect Ventilation Status in Selective Lung Ventilation: A Randomized Controlled Trial

**DOI:** 10.3389/fmed.2022.851395

**Published:** 2022-02-21

**Authors:** Tzu-Jung Wei, Ping-Yan Hsiung, Jen-Hao Liu, Tzu-Chun Lin, Fang-Tzu Kuo, Chun-Yu Wu

**Affiliations:** Department of Anesthesiology, National Taiwan University Hospital, Taipei, Taiwan

**Keywords:** personal protective equipment (PPE), lung ventilation, electronic stethoscop, COVID- 19, auscultation

## Abstract

Chest auscultation is the first procedure performed to detect endotracheal tube malpositioning but conventional stethoscopes do not conform to the personal protective equipment (PPE) protocol during the COVID-19 pandemic. This double-blinded randomized controlled trial evaluated the feasibility of using ear-contactless electronic stethoscope to identify endobronchial blocker established selective lung ventilation, simulating endobronchial intubation during thoracic surgery with full PPE. Conventional and electronic auscultation was performed without and with full PPE, respectively, of 50 patients with selective lung ventilation. The rates of correct ventilation status detection were 86 and 88% in the conventional and electronic auscultation groups (*p* = 1.00). Electronic auscultation revealed a positive predictive value of 87% (95% CI 77 to 93%), and a negative predictive value of 91% (95% CI 58 to 99%), comparable to the results for conventional auscultation. For detection of the true unilateral lung ventilation, the F1 score and the phi were 0.904 and 0.654, respectively for conventional auscultation; were 0.919 and 0.706, respectively for electronic auscultation. Furthermore, the user experience questionnaire revealed that the majority of participant anesthesiologists (90.5%) rated the audio quality of electronic lung sounds as comparable or superior to that of conventional acoustic lung sounds. In conclusion, electronic auscultation assessments of ventilation status as examined during thoracic surgery in full PPE were comparable in accuracy to corresponding conventional auscultation assessments made without PPE. Users reported satisfactory experience with the electronic stethoscope.

## Introduction

Chest auscultation of bilateral breath sounds is the first step in detecting endotracheal tube malpositioning. In view of the ongoing COVID-19 pandemic, clinicians performing endotracheal intubation for patients with COVID-19 infection experiencing respiratory distress must wear personal protective equipment (PPE) to prevent infection transmission (1–3). However, auscultation through conventional stethoscopy may breach the PPE protocol; thus, clinicians may have to abandon this procedure, a common physical examination in critical care. Telemedicine technology may offer a potential solution. For instance, electronic stethoscopes may provide adequate auscultation quality for practitioners complying with the PPE protocol because no earpiece is required and breath sounds can be transmitted through a speaker. However, this premise is difficult to investigate prospectively.

**Figure 1:**
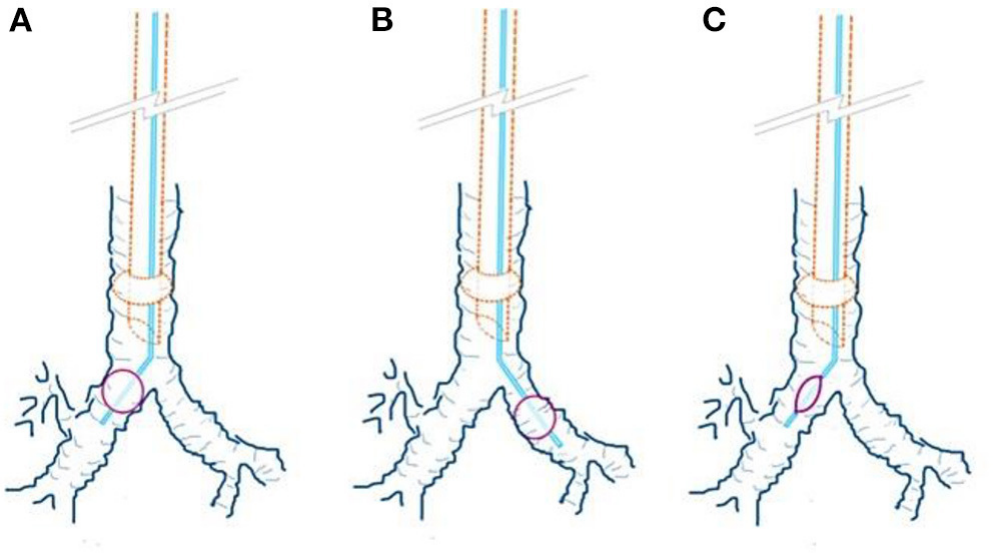
Endobronchial blocker (EB) position and ventilation statuses. **(A)** The right EB cuff was inflated; this simulates left endobronchial intubation. **(B)** The left EB cuff was inflated; this simulates right endobronchial intubation. **(C)** The EB cuff was not inflated; this simulates normal tracheal intubation.

Selective lung ventilation is mandatory for patients undergoing thoracic surgery, and it can be identified through conventional auscultation (4). Endobronchial blockers (EBs) can be used for this procedure and may simulate endobronchial intubation because asymmetric lung inflation is characteristic of both conditions (Figure 1).

Therefore, selective lung ventilation may be appropriate for safely testing the suitability of electronic auscultation for the PPE protocol. Through a double-blind randomized controlled trial, we compared the accuracy of electronic auscultation in full PPE and conventional auscultation performed without PPE to differentiate ventilation statuses during thoracic surgery.

## Method

### Patient Recruitment

**Figure 2:**
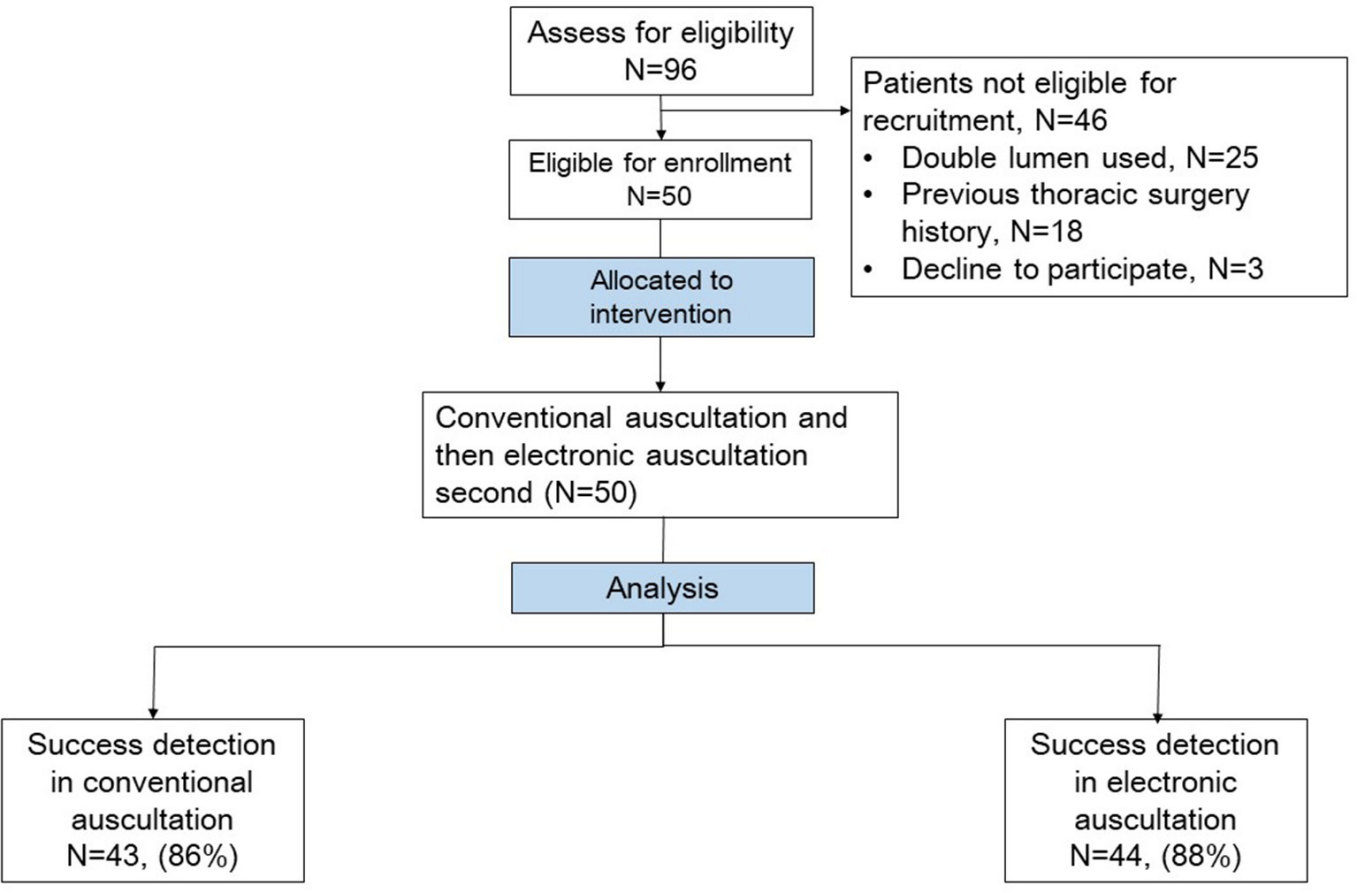
Patient enrolment.

A double-blind randomized controlled trial was conducted at a university hospital in Taipei, Taiwan. The study protocol was approved by the Research Ethics Committee of National Taiwan University Hospital and registered in the ClinicalTrials.gov protocol registration system (NCT04507958). Adult patients undergoing elective thoracoscopic surgery for lung tumor resection and using EBs for lung ventilation were recruited between September 2020 and May 2021. Patients with a history of lung surgery were excluded. All patients provided written informed consent the day before surgery to an investigator who was not involved in intraoperative care (Figure 2).

### Anesthesia, Intubation, and Lung Ventilation

General anesthesia was induced using a combination of 1–2 mcg/kg fentanyl, 0.2 mg of glycopyrrolate, 1–2 mg/kg propofol, and 0.2 mg/kg cisatracurium. The patients were intubated using an 8-mm cuffed endotracheal tube fixed at 20 cm at the incisor level. Depth of anesthesia was monitored and maintained the stable states between 40 to 60 with a Bispectral Index Monitor (BIS, Medtronic Co.) during the investigation and the operations. A Coopdech EB Tube (Daiken Medical Co., Ltd) was inserted to establish lung ventilation and to simulate endobronchial intubation. The EB position was confirmed by the attending anesthesiologist through fiberoptic bronchoscopy inspection, after which the state of lung ventilation was established based on a predefined randomization list. Three ventilation statuses were possible, namely the unilateral ventilation (inflation of the right or left EB cuff simulated endobronchial intubation of the left or right lung; Figures 1A,B) or bilateral ventilation (non-inflation of the EB cuff simulated normal endotracheal intubation; Figure 1C). During the auscultation test, the anesthesia machine and general monitor were partially covered to conceal the peak and mean airway pressure readings, capnography waveform, and pulse oximetry values because the participant anesthesiologists may have been able to identify one-lung ventilation on the basis of abnormal airway parameters rather than the auscultation (5). In addition, the EB cuff was concealed using a wrapped latex glove to ensure blind auscultation. Ventilation during auscultation was manually controlled by the primary care anesthesiologist with 20 cmH_2_O bagging pressure and respiratory rate of 10 to 15 times/min to maintain the SpO_2_ above 96% during the test.

### Preparation of Electronic Stethoscope and Auscultation

**Figure 3:**
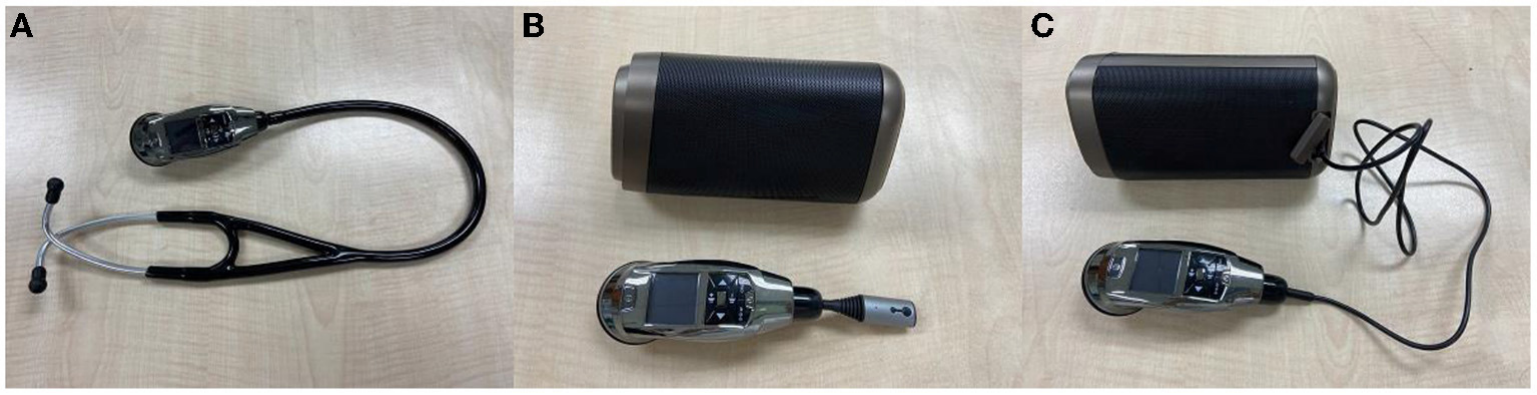
DS101 Electronic Stethoscope. **(A)**. DS101 electronic stethoscope with earpiece microphone. **(B)**. DS101 electronic stethoscope with Bluetooth transmitter for transmission of lung sound to external speaker. **(C)**. DS101 electronic stethoscope connected to external speaker by audio cable.

The DS101 electronic stethoscope (CARDIART, Taipei, Taiwan; Figure 3) was used in the present study. In typical conditions, the electronic stethoscope transmits lung sound by using an electret microphone placed in an earpiece, which operates similarly to a conventional stethoscope (Figure 3A). In the full PPE protocol, the earpiece microphone is inapplicable. However, the lung sound can be transmitted to the external speaker through wireless Bluetooth (Figure 3B) or by connecting an audio cable (Figure 3C). Because wireless lung sound transmission may be less cumbersome for clinicians in full PPE engaged in the task of endotracheal intubation, this method was selected. The Bluetooth pairing between the electronic stethoscope and the external speaker required ~1 to 2 min and was completed prior to the auscultation test.

**Figure 4:**
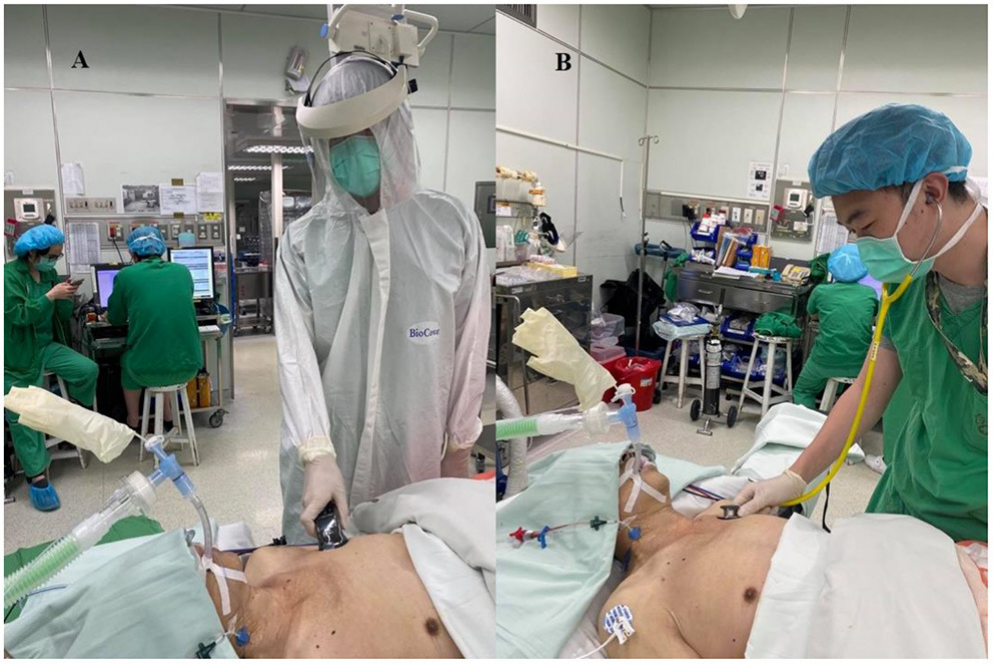
Illustrations of participant anesthesiologist setting. **(A)**. Illustration of full level C PPE (complying with the requirements of the Occupational Safety and Health Administration) in the electronic stethoscope group. **(B)**. Regular surgical scrubs and cap, not covering the face or ears in the conventional stethoscope group.

With reference to a related study into auscultation testing during endotracheal intubation, we invited as many anesthesiologists with diverse clinical experience as possible to exclude learning effects during the study (6). In total, 21 anesthesiologists participated in this trial. Anesthesiologists who did not participate in daily clinical care were randomly selected on the day of the test and randomly assigned to perform conventional or electronic auscultation. They waited in a separate room where they were unable to obtain information on the patient. After the selective lung ventilation state was established, they were separately brought into the operating room. Accordingly, the participant anesthesiologists were unable to communicate with each other. During the test, the anesthesiologist performing electronic auscultation wore full level C PPE according to the requirements of the US Occupational Safety and Health Administration, which included a disposable chemical resistance coverall, N95 mask, face shield, latex gloves, and boots (Figure 4A).

The electronic stethoscope provides three modes with distinct frequency ranges, namely bell mode (20–200 Hz), diaphragm mode (100–500 Hz), and wide mode (20–1,000 Hz). Because the use of a stethoscope is considered a basic physician skill, the three modes were introduced to the participant anesthesiologists and the anesthesiologists were not limited to a specific frequency mode during auscultation, despite the diaphragm mode being generally recommended. In addition, the electronic stethoscope has four organ settings (heart, lung, neck, and bowel) with various filters to eliminate ambient noise for optimal listening to the target organ. During the current study, the organ setting was restricted to the lung mode. The anesthesiologist performing conventional auscultation with a Littmann stethoscope (3M Health Care, St. Paul, MN, USA) wore regular surgical scrubs and a cap that did not cover the face or ears (Figure 4B).

Because the study was not conducted to investigate esophageal intubation, auscultation over the epigastrium was not performed. Moreover, the anesthesiologists were free to determine the order of auscultation of the left and right hemithorax because auscultation is considered a basic clinical skill. In consideration that clinicians in the PPE protocol may require more time to perform electronic auscultation (due to the visual and auditory barriers of the PPE), the auscultation test time was set to 90 s in both study groups and this was approximately twice the reported median time in conventional auscultation to detect endobronchial intubation (7). Furthermore, a clinical research assistant who was blinded to patient characteristics and who was not responsible for clinical work completed the associated study records. Participant anesthesiologists were not made aware of true lung ventilation states during the entire investigation period to avoid potential learning effects.

After trial completion, anesthesiologists who had used the electronic stethoscope were asked to provide feedback on the electronic auscultation. The three metrics were the overall audio quality (vs. conventional stethoscope), difficulty of electronic auscultation, and lung sound quality of the electronic auscultation. A conventional stethoscope's noise insulation may enhance clinicians' concentration during conventional auscultation. To obtain similar noise cancelation, the manufacturers of electronic stethoscopes implement various digital filters to remove unwanted frequency and noise. For instance, the DS101 electronic stethoscope includes different organ modes. Therefore, the participants were asked to evaluate the audio quality of the electronic stethoscope in comparison with conventional stethoscopes. Specifically, they were asked whether the electronic auscultation was superior or inferior to conventional auscultation based on the overall auscultation clarity and their personal preference between the devices for future auscultation for selective lung ventilation.

The difficulty metric was related to the convenience of the interface and the user operating experience. Accordingly, the participant anesthesiologists were asked to rate the difficulty of operating the device during the auscultation on a scale of 0 to 10 (0: not difficult at all; 6: occasional difficulty during the test period; 10: extremely difficult during the test period). Furthermore, because the heart sound and lung sound are 2 major body sound signals from the chest region and often interfere with each other during auscultation, manufacturers provide unique algorithms for the separation of heart and lung sound (8). Therefore, lung sound quality in the questionnaire referred to the quality of the heart and lung sound separation during auscultation (0–10; 0: heart sound was comparable to or more obvious than lung sound with frequent heart sound interference during auscultation; 6: partial sound separation with occasional heart sound interference during auscultation; 10: complete sound separation without detectable heart sound during lung sound auscultation).

### Statistical Analysis

The primary outcome was the ability to detect the lung ventilation state, which referred to endotracheal tube positioning at tracheal or bronchial sites. One study reported a success rate of EB positioning by conventional auscultation of ~90% (4). In another study, auscultation detected the endotracheal tube in the tracheal position at an average rate of 92.5% but at the endobronchial position at an average rate of 85% under optimal conditions, for a difference of ~7% (6). On the basis of these studies, a sample size of 41 patients was determined necessary for achieving a 90% detection rate with a non-inferiority margin of 7%, an alpha value of 0.05, and at a power of 0.8 to indicate comparable accuracy between the two auscultation methods. In consideration of potential attrition, 50 patients were enrolled. All proportions were tested using a proportional *z* test or Fisher exact test to compare the accuracy of conventional and electronic auscultation and to compare the assignments of anesthesiologists with diverse clinical experience. Furthermore, McNemar's test was used to compare the accuracy of conventional and electronic auscultation. The confusion matrices including the F1 score and the Matthews correlation coefficient (phi) were also calculated to evaluate the performance of the conventional and electronic auscultation on detection of true unilateral lung ventilation. In addition leave-one-out cross validation (LOOCV) was also performed and the average performance of 10 times of the LOOCV was calculated to avoid overfitting the model (9). Interrater agreement was analyzed by calculating the *k* coefficient. Statistical analysis was performed using PASS Sample Size Software 2021 (NCSS LLC, Kaysville, UT, USA) and MedCalc Statistical Software version 20 (MedCalc Software Ltd., Ostend, Belgium).

## Results

In this study, 96 patients undergoing thoracoscopic lung tumor resection were initially included, 46 of which were excluded and one withdraw the consent, as shown in Figure 2. The characteristics of the 50 patients are summarized in Table 1.

**Table 1:** Patient characteristics.

	**Number of patients**
Age (years)	60 (16)
Height (cm)	163 (10)
Weight (kg)	65.6 (12.1)
Body mass index (kg/m^2^)	24.7 (3.9)
Chronic obstructive pulmonary disease (*n*; %)	10 (20%)
ASA classification (*n*)	
I	5
II	24
III	19
IV	2

*Data are presented as numbers (percentages) or means ± standard deviations (ranges)*.

Ten patients (20%) presented with chronic obstructive pulmonary disease. The average number of auscultations for each anesthesiologist was 2.4 ± 2.3 vs. 2.4 ± 1.5 for conventional vs. electronic auscultation (*P* = 1.000). No Bluetooth signal interference or disruption occurred during auscultation tests.

Among the 50 paired tests, 16, 19, and 15 involved the right EB cuff being inflated (simulating left endobronchial intubation), left EB cuff being inflated (simulating right endobronchial intubation), and EB cuff not being inflated (bilateral lung ventilation), respectively. Electronic auscultation revealed that ventilation status was correctly detected 88% of the time with a sensitivity of 97%, specificity of 67%, positive predictive value of 87% (95 CI 77 to 93%), and negative predictive value of 91% (58 to 99%). The conventional auscultation results were similar (Table 2). For detection of the true unilateral lung ventilation, the F1 score and the phi were 0.904 and 0.654, respectively for conventional auscultation; were 0.919 and 0.706, respectively for electronic auscultation (Table 2). Furthermore, the average performance of the LOOCV revealed the F1 score of 0.900 and phi of 0.647 for the conventional auscultation to detect unilateral lung ventilation; the F1 score of 0.919 and phi of 0.710 for the for the electronic auscultation to detect unilateral lung ventilation. These results were similar to the metrics calculated from the whole cohort. Details of the LOOCV were listed in the Supplementary Material.

**Table 2:** Identification of ventilation statuses through conventional and electronic auscultation.

	**True ventilation state**				**True ventilation state**		
**Conventional**	**Unilateral**	**Bilateral**	**Total**	**Electronic**	**Unilateral**	**Bilateral**	**Total**
Correct	33	10	43	Correct	34	10	44
Incorrect	2	5	7	Incorrect	1	5	6
Total	35	15	50	Total	35	15	50
Chi-square comparison (*P* = 1.000)	Number correct 43 (86%)				Number correct 44 (88%)		
Sensitivity to unilateral	94% (81–99%)	PPV	87 (77–93)	Sensitivity to unilateral	97% (85–100%)	PPV	87 (77–93)
Specificity to unilateral	67% (38–88%)	NPV	83 (55–95)	Specificity to unilateral	67% (38-88%)	NPV	91 (58–99)
F1 score	0.904	phi	0.654	F1 score	0.919	phi	0.706

Seven conventional auscultation tests were failed; the true lung ventilation states comprised two cases of one-lung ventilation and 5 of two-lung ventilation. Similarly, 6 electronic auscultation tests were failed; the true lung ventilation states comprised one case of one-lung ventilation and 5 of two-lung ventilation. Therefore, most of the failures (76.9%) were due to the uncertainty of bilateral symmetric lung sound regardless of the auscultation method. The McNemar test indicated a non-significant difference between electronic and conventional auscultation with a 2% difference (95% CI −12.4 to 8.4%; *P* = 1.000; Table 3).

**Table 3:** Correlation of lung ventilation detection through conventional and electronic auscultation.

		**Conventional aucustation**		
		**Correct**	**Incorrect**	**Total**
Electronic auscultation	Correct	40	4	44 (88%)^a^
	Incorrect	3	3	6 (12%)
	Total	43 (86%)^a^	7 (14%)	

*Mcnemar test result P = 1.00*.

a *Correct rate*.

In addition, the interrater agreement between electronic and conventional auscultation was excellent, as indicated by a weighted *k* coefficient of 0.81 (95% CI 0.68 to 0.94). Overall, the anesthesiologists were satisfied with the electronic stethoscope (Table 4).

**Table 4:** User satisfaction with the electronic stethoscope.

**Questionnaire**	**Result**
Audio quality between the two stethoscopes (*n*; %) Electronic is preferred Conventional is preferred Comparable	*N* = 21 5 (23.8%) 2 (9.5%) 14 (66.7%)
Difficulty of using the electronic stethoscope (0–10)	2.6 ± 1.8 (0–7)
Lung sound quality of the electronic stethoscope (0–10)	7.8 ± 1.6 (5–10)

*Data are presented as numbers (percentages) or means ± standard deviations (ranges)*.

Five anesthesiologists (23.8%) reported that they preferred it over the conventional stethoscope, and 14 (66.7%) reported that the 2 stethoscopes were comparable. The mean ± standard deviation of difficulty of use and lung sound quality with regard to electronic stethoscopy in full PPE were 2.6 ± 1.8 and 7.8 ± 1.6, respectively (Table 4).

## Discussion

The rate of accurate detection of ventilation status through electronic auscultation was favorable even when the PPE protocol was followed. The advantages of using lung ventilation during thoracic surgery for the simulation of endotracheal tube malpositioning are 2-fold. First, it achieves asymmetric lung ventilation without additional risk to the patient; second, the EB balloon can be covered to conceal the ventilation status, thereby allowing a blind test to be conducted. However, Ramsingh et al. reported a lower accuracy rate of 66% with regard to the detection of endobronchial intubation through conventional auscultation performed without PPE (5). This may be due to several factors. First, substantial gas inflow from the lung opposite the side of the endobronchial intubation may have occurred because the endotracheal tube cuff only partially obstructed the main bronchus; however, the EB can prevent most gas inflow from the opposite lung. Second, the pressure and respiratory rate for manual bagging was not controlled in the study by Ramsingh et al. The current study performed manual ventilation with a bagging pressure of 20 cmH_2_O and a respiratory rate of 10 to 15 times/min. When PPE protocol is not followed, clinicians can perform examination adequately without visual obstacles, and concentrating on auscultation is easier because conventional stethoscopes provide noise insulation. However, negative cumulative effects such as thermal stress, limitations on hearing and vision, and restriction of movement under PPE protocol greatly impede examination and auscultation (1). Our results revealed that these physical limitations of the PPE protocol may be attenuated by using the electronic stethoscope. In addition, two previous preliminary studies have indicated that a computerized analysis of lung sounds obtained through electronic stethoscopy was able to detect esophageal or endobronchial intubation accurately (10, 11). With advances in deep learning, improvements in the accuracy of electronic auscultation are expected. Furthermore, user satisfaction with the electronic stethoscope was high under PPE protocol. This finding accords with a report that electronic stethoscopes were comparable or superior to acoustic devices in 95% of examinations (12).

The current guideline for managing the airway of patients with COVID-19 includes the use of video laryngoscopy and capnography for the basic verification of endotracheal intubation (13). The clinician in charge of intubation is advised not to lose sight of the endotracheal tube on the screen and to pass the cuff 1 to 2 cm below the vocal cords to avoid endobronchial intubation; the continuous waveform of capnography can be used to confirm the intubation (13). The guideline also highlights the difficulty of auscultation of lung sound in the PPE protocol. However, maintenance of adequate vision during intubation can be challenging for clinicians, particularly in high-tension environments and with the physical barriers of PPE. In addition, instant capnography check does not guarantee the avoidance of endobronchial intubation (14–16). For instance, hypoxemia may occur later, (15) and capnography measurements may not change in endobronchial intubation (14, 16). Nevertheless, our results indicated that most of the auscultation test failures (76.9%) occurred because the participant anesthesiologist was unable to confirm bilateral symmetric lung sound, regardless of the auscultation method. This result reflects the difficulty of distinguishing subtle lung sound differences between the two lungs, and ambient noise precludes accurate auscultation. Although auscultation is not the gold standard method for confirmation of endotracheal tube position, it is a procedure familiar to care providers, and electronic auscultation can be performed rapidly even in the full PPE protocol. Moreover, the capnography and peak airway pressure measurements were concealed during the study to ensure the validity of the values detected during the auscultations. Furthermore, a study revealed that clinicians could determine the endotracheal tube position in almost 100% of cases by using a combination of conventional auscultation and endotracheal tube insertion depth (6). In the current study, the EB was used to simulate endobronchial intubation; thus, endotracheal tube insertion depth was regarded as unknown to the anesthesiologists. Accordingly, the accuracy of the use of electronic auscultation combined with airway parameters and observation of endotracheal tube depth in detecting lung ventilation states may be higher than reported in the current study. Therefore, we consider the use of electronic stethoscopy as having potential clinical benefits in first-line uses in the PPE protocol.

Ultrasound is theoretically better for confirming endotracheal tube position than is conventional auscultation with regard to higher accuracy (5) and it is also feasible under PPE protocol. Accordingly, this approach has been repeatedly proposed in recent commentaries on COVID-19 patients (17–19). However, the widespread application of ultrasound during the ongoing COVID-19 pandemic has several major drawbacks. First, training and the dissemination of knowledge on adequate ultrasound examination is likely to be less comprehensive given that frontline health care personnel are preoccupied with managing the heavy burden of COVID-19 clinical care. This is particularly relevant in countries with fewer medical resources. By contrast, auscultation is regarded as a basic technique by health care personnel including physicians and nurses. Our results consistently indicated a high accuracy rate for electronic auscultation in identifying selective lung ventilation and the participant anesthesiologists rated a low difficulty in the usage of electronic stethoscopy with a full PPE protocol. Second, the price of electronic stethoscopes is considerably lower than that of ultrasound devices. For instance, the price of the DS101 electronic stethoscope used in this study is less than half that of a common point-of-care ultrasound system. Third, the transport of an ultrasound device in and out of a negative pressure isolation ward may increase the risk of infection transmission because the microdroplets produced by endotracheal intubation may linger in the environment (20). By contrast, electronic stethoscopes are easily portable, and all their components can be sanitized using 75% alcohol or a comparable disinfectant (21).

During thoracic surgery, lung ventilation is usually achieved using a fiberoptic bronchoscope to confirm the proper position of the EB and the double-lumen endotracheal tube. However, the use of a fiberoptic bronchoscope is associated with contagious transmission risk (22). Accordingly, concerns regarding the use of bronchoscopy have been highlighted in guidelines for thoracic surgery during the COVID-19 pandemic (23–26). Auscultation for lung ventilation is not mentioned in most guidelines, but one recent guideline suggested avoiding auscultation for selective lung ventilation because of the difficulty of using a stethoscope in full PPE (25). However, in this study, auscultation with the ear-contactless electronic stethoscope enabled the identification of the position and inflation status of the EB with satisfactory accuracy over a short period (90 s). This result is also consistent with a study indicating a high accuracy rate for EB position confirmation through conventional auscultation during thoracoscopic surgery (4). Accordingly, our findings may serve as a reference for selective lung ventilation protocol for patients with COVID-19 undergoing thoracic surgery. In addition, we observed favorable sensitivity, positive predictive values and F1 score of the auscultation to detect unilateral lung ventilation. However, the specificity and the phi correlation coefficient were relatively low and the range of negative predictive value was wide. These results indicated that auscultation may achieve better result to detect true positive (unilateral lung ventilation) but may fall short in detecting true negative (two lungs ventilation). Because unilateral lung ventilation is mandatory and is intentionally established during thoracic surgery, the implementation of electronic auscultation in thoracic surgery when the PPE protocol is required may be particularly useful. Furthermore, the obstructive lung disease is a common comorbidity among patients undergoing thoracic surgery and accordingly we observed that a considerable number of participants (20%) had obstructive pulmonary disease in the present study. The intensity and distribution of lung sounds are profound altered in patients with obstructive lung disease (27) and this may hamper correct auscultation. Nevertheles,. there were accumulating studies focusing on the clinical validity of electronic auscultation for the obstructive lung disease (28, 29) and thus this may indicate additional values of implementing electronic auscultation during thoracic surgeries.

This study has several limitations. First, the sample size was small and only one medical center was involved. In consideration of this issue, the LOOCV was performed to avoid overfitting which was relatively common in studies with smaller sample size and we observed similar results after averaging the LOOCV. However, future research with a larger sample size is warranted. Second, auscultation was performed for the confirmation of already established lung ventilation. One can expect a more challenging situation for electronic auscultation in full PPE to establish selective lung ventilation because this procedure takes a longer time than does confirmation alone. Third, we set a longer auscultation time limit in this study because of clinician performance was often hampered in PPE protocol (1). However, each participant anesthesiologists in both study groups conducted the auscultation task within one min. It may be possible to reach better results in shorter test time and this may be experimented in future researches.

In conclusion, electronic auscultation in full PPE was non-inferior to conventional auscultation without PPE for the confirmation of selective lung ventilation in patients undergoing thoracic surgery. Further investigation exploring the actual utility of an electronic stethoscope for instant lung sound examination after the endotracheal intubation of patients with COVID-19 is warranted.

## Data Availability Statement

The original contributions presented in the study are included in the article/Supplementary Material, further inquiries can be directed to the corresponding author/s.

## Ethics Statement

The studies involving human participants were reviewed and approved by The Research Ethics Committee of National Taiwan University Hospital, Taipei, Taiwan. The patients/participants provided their written informed consent to participate in this study.

## Author Contributions

T-JW and C-YW: conceived and designed the study. J-HL and C-YW: obtained ethical approval. T-JW, P-YH, T-CL, F-TK, and C-YW: collected clinical data. P-YH: analyzed the data. C-YW: wrote the first draft of the manuscript. All authors reviewed and approved the final version of the manuscript for submission.

## Funding

This work was supported by research financial support and sponsorship from the Ministry of Science and Technology, Taipei, Taiwan (109-2327-B-002 -010).

## Conflict of Interest

The authors declare that the research was conducted in the absence of any commercial or financial relationships that could be construed as a potential conflict of interest.

## Publisher's Note

All claims expressed in this article are solely those of the authors and do not necessarily represent those of their affiliated organizations, or those of the publisher, the editors and the reviewers. Any product that may be evaluated in this article, or claim that may be made by its manufacturer, is not guaranteed or endorsed by the publisher.
