# Supplementary material for: Use of Electronic Auscultation in Full Personal Protective Equipment to Detect Ventilation Status in Selective Lung Ventilation: A Randomized Controlled Trial
Source: Front Med (Lausanne). 2022 Feb 21;9:851395. doi: 10.3389/fmed.2022.851395 (PMC8899469; doi:10.3389/fmed.2022.851395)
Supplement: Supplementary file 1 [file Data_Sheet_1.docx]

| **Table 1.** Leave-one-out cross validation | | | | | | |
| --- | --- | --- | --- | --- | --- | --- |
|  | Sensitivity | Specificity | Accuracy | F1 score |  | MCC |
|  |  |  |  | Unilateral | Bilateral |  |
| Conventional | 66.50% | 93.93% | 85.56% | 90.04% | 73.71% | 0.647 |
| Electronic | 66.50% | 97.44% | 88.00% | 91.86% | 77.13% | 0.710 |

MCC: Matthews correlation coefficient

| **Table 2.** Conventional auscultation leave-one-out cross validation | | | | | | | | | | | |
| --- | --- | --- | --- | --- | --- | --- | --- | --- | --- | --- | --- |
|  | True ventilation state | | | |  | Sensitivity | Specificity | Accuracy | F1 score |  | MCC |
|  | Unilateral | | Bilateral |  | Total |  |  |  | Unilateral | Bilateral |  |
| SampleNo. | Correct | Incorrect | Correct | Incorrect |  |  |  |  |  |  |  |
| 1 | 30 | 4 | 9 | 2 | 45 | 69.23% | 93.75% | 86.67% | 90.91% | 75.00% | 0.664 |
| 2 | 29 | 5 | 9 | 2 | 45 | 64.29% | 93.55% | 84.44% | 89.23% | 72.00% | 0.623 |
| 3 | 29 | 5 | 9 | 2 | 45 | 64.29% | 93.55% | 84.44% | 89.23% | 72.00% | 0.623 |
| 4 | 30 | 5 | 9 | 1 | 45 | 64.29% | 96.77% | 86.67% | 90.91% | 75.00% | 0.680 |
| 5 | 30 | 4 | 9 | 2 | 45 | 69.23% | 93.75% | 86.67% | 90.91% | 75.00% | 0.664 |
| 6 | 30 | 4 | 9 | 2 | 45 | 69.23% | 93.75% | 86.67% | 90.91% | 75.00% | 0.664 |
| 7 | 29 | 5 | 9 | 2 | 45 | 64.29% | 93.55% | 84.44% | 89.23% | 72.00% | 0.623 |
| 8 | 29 | 5 | 9 | 2 | 45 | 64.29% | 93.55% | 84.44% | 89.23% | 72.00% | 0.623 |
| 9 | 28 | 5 | 10 | 2 | 45 | 66.67% | 93.33% | 84.44% | 88.89% | 74.07% | 0.640 |
| 10 | 30 | 4 | 9 | 2 | 45 | 69.23% | 93.75% | 86.67% | 90.91% | 75.00% | 0.664 |
| MCC: Matthews correlation coefficient | | | | | | | | | | | |

| **Table 3.** Electronic auscultation leave-one-out cross validation | | | | | | | | | | | | |
| --- | --- | --- | --- | --- | --- | --- | --- | --- | --- | --- | --- | --- |
|  | True ventilation state | | | |  |  | Sensitivity | Specificity | Accuracy | F1 score |  | MCC |
|  | Unilateral | | Bilateral |  | | Total |  |  |  | Unilateral | Bilateral |  |
| Sample No. | Correct | Incorrect | Correct | Incorrect | |  |  |  |  |  |  |  |
| 1 | 31 | 4 | 9 | 1 | | 45 | 69.23% | 96.88% | 88.89% | 92.54% | 78.26% | 0.721 |
| 2 | 30 | 5 | 9 | 1 | | 45 | 64.29% | 96.77% | 86.67% | 90.91% | 75.00% | 0.680 |
| 3 | 31 | 5 | 9 | 0 | | 45 | 64.29% | 100.00% | 88.89% | 92.54% | 78.26% | 0.744 |
| 4 | 30 | 5 | 9 | 1 | | 45 | 64.29% | 96.77% | 86.67% | 90.91% | 75.00% | 0.680 |
| 5 | 32 | 4 | 9 | 0 | | 45 | 69.23% | 100.00% | 91.11% | 94.12% | 81.82% | 0.784 |
| 6 | 31 | 3 | 10 | 1 | | 45 | 76.92% | 96.88% | 91.11% | 93.94% | 83.33% | 0.778 |
| 7 | 30 | 5 | 9 | 1 | | 45 | 64.29% | 96.77% | 86.67% | 90.91% | 75.00% | 0.680 |
| 8 | 30 | 5 | 9 | 1 | | 45 | 64.29% | 96.77% | 86.67% | 90.91% | 75.00% | 0.680 |
| 9 | 29 | 5 | 9 | 1 | | 44 | 64.29% | 96.67% | 86.36% | 90.63% | 76.92% | 0.695 |
| 10 | 31 | 5 | 8 | 1 | | 45 | 61.54% | 96.88% | 86.67% | 91.18% | 72.73% | 0.662 |
| MCC: Matthews correlation coefficient | | | | | | | | | | | | |
